# Comparison of feature tracking and harmonic phase imaging for strain measurement on cardiovascular magnetic resonance in pediatric cancer survivors

**DOI:** 10.1186/1532-429X-16-S1-P334

**Published:** 2014-01-16

**Authors:** Jimmy C Lu, James A Connelly, Lili Zhao, Prachi P Agarwal, Adam L Dorfman

**Affiliations:** 1 Pediatrics and Communicable Diseases, University of Michigan, Ann Arbor, Michigan, USA; 2 Radiology, University of Michigan, Ann Arbor, Michigan, USA; 3 Biostatistics, University of Michigan, Ann Arbor, Michigan, USA

## Background

Pediatric cancer survivors after anthracycline therapy are at risk for left ventricular systolic dysfunction. Strain may be a more sensitive marker than left ventricular ejection fraction (LVEF). Feature tracking (FT) is a promising technique for strain measurement on cardiovascular magnetic resonance (CMR) images, but has limited validation vs. the gold standard of harmonic phase (HARP) imaging analysis. This study aimed to compare strain by FT and HARP on CMR images in pediatric cancer survivors.

## Methods

Twenty-six patients (20.2 ± 5.6 years old) underwent CMR at least 5 years after completing anthracycline therapy. Short-axis myocardial tagged images and steady state free precession images were analyzed by HARP and FT for circumferential and radial strain at the base, midventricle and apex. FT measurements were performed by two blinded readers for intraobserver and interobserver variability.

## Results

LVEF more closely correlated with global circumferential strain by FT (r = -0.63, p = 0.0005) than by HARP (r = -0.39, p = 0.05). Midventricular circumferential strain by FT and HARP did not significantly differ (mean difference -1.2, limits of agreement -7.7 to 5.2). Midventricular circumferential strain by FT closely approximated global circumferential strain by FT (r = 0.86, p < 0.0001). Radial strain by FT was markedly underestimated relative to HARP, particularly at higher values of radial strain. Intraobserver and interobserver reproducibility was excellent for FT global circumferential strain (coefficients of variation 4.8%, 3.2%), but poor for FT global radial strain (32%, 25%).

## Conclusions

Circumferential strain by FT is a reliable and reproducible measure of myocardial deformation in patients status post anthracycline therapy, while radial strain measurements are limited. Further studies are necessary to evaluate potential relation to long-term outcomes.

## Funding

This study was supported by a University of Michigan Cancer Research Committe Award.

